# Personalized Delivery of Probiotics and Prebiotics via 3D Food Printing

**DOI:** 10.3390/metabo15110744

**Published:** 2025-11-17

**Authors:** Jiyoung Yu

**Affiliations:** Department of Convergence, Superstar College, Jeonju University, 303 Cheonjam-ro, Wansan-gu, Jeonju-si 55069, Republic of Korea; jiyoung@jj.ac.kr; Tel.: +82-063-220-2264

**Keywords:** 3D food printing, probiotics, prebiotics, synbiotics, personalized diets

## Abstract

Personalized nutrition aims to optimize health by addressing interindividual differences in metabolism, microbiota composition, and dietary responses. Modulating the gut microbiota through probiotics, prebiotics, and synbiotics is promising, yet conventional systems such as capsules or fermented foods offer limited control over dosage, release kinetics, and microbial viability. These formats often cause 2–4 log reductions in viable counts during processing and gastrointestinal transit, underscoring the need for advanced delivery technologies. Three-dimensional (3D) food printing enables digital design of edible matrices with programmable geometry and composition to enhance microbial protection and controlled release. Coaxial and gel-in-gel architectures have retained over 90–96% of probiotic cells after printing and 80–85% after simulated digestion. Synbiotic formulations combining probiotics with fructooligosaccharides or whey protein achieve 98–99% survival and stability for 35 days. This review summarizes advances in formulation, encapsulation, and printing strategies, highlighting how 3D food printing uniquely overcomes challenges of viability, release control, and personalized dosage in microbiota-based nutrition.

## 1. Introduction

Nutritional requirements are different for each individual and are substantially influenced by individual lifestyle and environmental factors. Nutritional requirements depend on the life cycle, sex, occupation, physical activity level, lifestyle habits, and geographic location. Infants require breast milk or specialized formulas enriched in easily digestible proteins, long-chain polyunsaturated fatty acids, and immunomodulatory components to support development. During adolescence and early adulthood, rapid growth is accompanied by increased demands for energy and protein, whereas older adults require nutrient-dense diets to mitigate sarcopenia and reduced metabolic efficiency [1]. These differences have traditionally formed the foundation of dietary guidelines across the human lifespan. Recent evidence has shown that individuals exhibit highly variable metabolic and glycemic responses to identical foods or nutrients [2,3,4]. These findings reveal personalized nutrition as a strategy to optimize health.

Variability in nutritional responses between individuals is strongly influenced by the gut microbiome, which mediates host–diet interactions. Variability in the composition and abundance of microbial communities among individuals explains why identical nutrient intake can yield divergent outcomes [5]. The gut microbiome has constituted a vast community of bacteria, archaea, fungi, and viruses, whose collective gene pool exceeds that of the human genome. These microorganisms contribute not only to digestion, energy harvest, and vitamin synthesis but also to immune maturation, epithelial barrier integrity, and defense against pathogens. Short-chain fatty acids, such as acetate, propionate, and butyrate, regulate glucose and lipid metabolism, appetite, and epithelial health, so these metabolites influence all aspects of metabolism overall [6,7,8,9]. Such evidence has established the gut microbiome as a distinct metabolic and immunological organ. Accordingly, interindividual differences in the composition and activity of the gut microbiome help explain the marked variability in dietary responses [10]. Recent studies have demonstrated that individuals consuming similar meals can exhibit markedly different postprandial metabolic responses—particularly in glucose and triglyceride levels—partly influenced by inter-individual differences in gut microbiota composition and metabolic regulation [2,3]. Dysbiosis, defined as an imbalance in microbial composition or function, has been consistently associated with a wide spectrum of diseases. Alterations in the gut microbiome have been linked to obesity, type 2 diabetes, irritable bowel syndrome, colorectal cancer, and autoimmune diseases, as demonstrated by recent human studies [11,12,13,14,15]. While the precise causal relationship remains under investigation, animal and human studies demonstrate that restoring and maintaining microbial balance has beneficial health effects. Consequently, strategies to modulate the gut microbiome are emerging as an important component of preventive and therapeutic nutrition.

**Figure 1:**
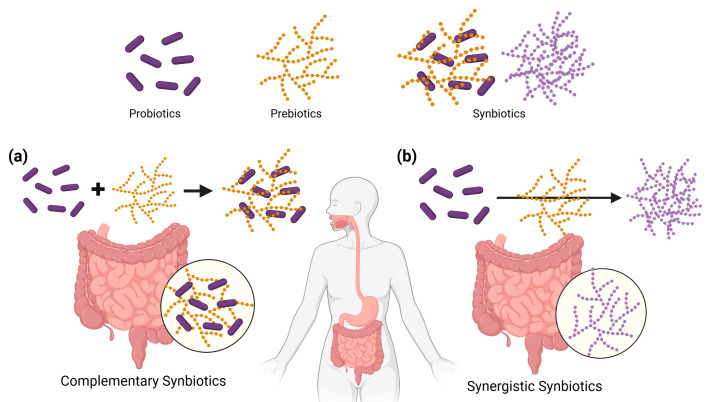
Probiotics, prebiotics, and synbiotics, and two synbiotic design strategies; (**a**) Complementary synbiotics and (**b**) Synergistic synbiotics.

Modulating the gut microbiome for disease prevention and treatment is a rapidly growing field in personalized nutrition. Advances in sequencing technologies and bioinformatics have enabled detailed profiling of individual microbiota, allowing the identification of depleted or enriched taxa. Such information can be used to design targeted dietary strategies aimed at restoring ecological balance [16]. The core components of personalized microbiome-based nutrition are probiotics, prebiotics, and synbiotics. According to the International Scientific Association for Probiotics and Prebiotics (ISAPP), probiotics are defined as ‘live microorganisms that, when administered in adequate amounts, confer a health benefit on the host [17]. Numerous clinical studies have demonstrated their efficacy in strengthening gut barrier function, modulating immune responses, reducing gastrointestinal (GI) infections, and improving metabolic parameters [18,19,20,21,22]. Prebiotics, in contrast, are selectively fermented substrates such as inulin, fructo-oligosaccharides, or resistant starches that promote the growth or activity of beneficial microbes. For example, outcomes by modulating the composition of the microbiota and enhancing short-chain fatty acid production [23]. According to the ISAPP, synbiotics are defined as mixtures comprising live microorganisms and substrates selectively utilized by host microorganisms that confer a health benefit on the host (Figure 1) [24] Importantly, because the microbial ecosystem is unique to each host, the selection of appropriate strains and substrates must be individualized [25]. For example, one individual may benefit from prebiotics that stimulate *Bifidobacterium* growth, whereas another may require interventions focused on lactobacilli, or a tailored combination to restore microbial balance. Personalization of microbiome interventions is therefore increasingly recognized as a key pathway toward precision nutrition. Recent advances in microbiome research have emphasized that individualized modulation of the gut microbiota requires not only biological understanding but also technological means to translate these insights into practical dietary formats. Three-dimensional food printing provides such a bridge by enabling the integration of probiotics, prebiotics, and bioactive substrates into spatially designed food structures. Through programmable architectures, 3D printing allows localized protection, targeted release, and tailored dosing of microbial and nutritional components—transforming microbiome modulation concepts into physically personalized nutrition solutions.

Although extensive research has been conducted on personalized nutrition using microbiome information, conventional delivery formats of probiotics and prebiotics, such as capsules, powders, or fortified yogurts, offer limited opportunities for customization. Dosages are typically standardized, formulations are fixed, and release kinetics and G transit remain challenging [26,27]. A recently emerging technology that potentially addresses these limitations is 3D food printing, an additive manufacturing method that deposits edible materials layer by layer according to a digital design, producing food with customized shapes and textures [28]. Unlike conventional encapsulation or coating technologies, 3D printing enables digital control over formulation and structure, allowing the creation of multilayered, compartmentalized, and structurally complex constructs suitable for personalization. Nutrient composition, dosage, texture, and release profiles can be digitally adjusted to meet individual requirements [29,30]. Probiotics and prebiotics can be loaded within protective matrices, combined with complementary bioactive compounds, or arranged in structures designed for targeted release in specific GI regions [31]. Therefore, 3D food printing serves as a practical bridge connecting microbiome research to personalized nutrition, enabling the translation of mechanistic insights into actionable dietary strategies.

The objective of this review is to examine personalized nutrition strategies centered on probiotics, prebiotics, and 3D food printing. The review summarizes current knowledge of how probiotics and prebiotics modulate the gut microbiome and emphasizes the limitations of conventional delivery systems. Furthermore, it describes how 3D food printing can specifically address unresolved issues in probiotic and prebiotic delivery—such as instability during processing, loss of viability under gastrointestinal stress, and lack of patient-specific dosage control—by enabling customizable architectures and programmable release profiles. It focuses on formulation strategies, process considerations, and synbiotic applications. Future directions for research and practical implementation are presented to advance personalized microbiome and diet planning using 3D food printing.

## 2. Diet, Probiotics, and Prebiotics in Microbiota Modulation

### 2.1. Diet in Microbiota Modulation

Diet is a fundamental determinant of gut microbiome composition, diversity, and function, with effects observable over both short and long timescales. Controlled feeding studies have demonstrated that macronutrient composition can alter microbial communities within days. Animal-based diets enrich bile-tolerant genera such as *Bilophila* and *Bacteroides* while reducing fiber-degrading taxa, including *Roseburia* and *Eubacterium* [32]. In contrast, plant-based diets rapidly increase saccharolytic species and the abundance of genes encoding glycoside hydrolases, reflecting enhanced capacity for polysaccharide fermentation. These alterations are not limited to taxonomic changes but also extend to functional adaptations [33,34]. Specifically, animal-based diets modulate microbial transcriptional activity to favor bile acid metabolism, whereas plant-based diets promote carbohydrate fermentation and short-chain fatty acid (SCFA) pathways [32,35]. Long-term dietary patterns further reinforce these differences. Populations adhering to Mediterranean-style diets, rich in plant-based foods and olive oil, exhibit favorable shifts in gut microbiota, including increased abundance of *Faecalibacterium prausnitzii* and other butyrate-producing bacteria, together with altered bile acid metabolism [36]. By contrast, Western diets rich in saturated fats, refined sugars, and processed foods are associated with reduced microbial diversity. By contrast, Western diets rich in saturated fats, refined sugars, and processed foods are associated with reduced microbial diversity. These divergences may contribute to the protective effects of Mediterranean-style diets against metabolic and inflammatory disorders, as supported by controlled intervention and cohort studies [36,37,38,39].

Indigestible polysaccharides, collectively referred to as dietary fiber, represent significant class of dietary constituents. These fibers generally reach the colon largely undigested, where they function as primary substrates for microbial fermentation. The fermentation products, namely the SCFAs acetate, propionate, and butyrate, have been reported to confer diverse physiological benefits. Acetate is involved in cholesterol metabolism and appetite regulation. Propionate contributes to hepatic gluconeogenesis and satiety pathways. Butyrate functions as the primary energy source for colonocytes and acts as a histone deacetylase inhibitor, thereby influencing gene expression, epithelial barrier integrity, and immune tolerance [40,41]. The concentration and profile of SCFAs are determined by both the types of fibers consumed and the microbial taxa present, indicating that dietary composition and baseline microbiota jointly determine metabolic outcomes. For example, resistant starch supplementation increases the abundance of *Ruminococcus bromii* and *Eubacterium rectale*, taxa specialized in resistant starch degradation, and this enhances butyrate production. In contrast, low-fiber diets reduce SCFA concentrations and promote mucin-degrading organisms such as *Akkermansia* under fiber-deficient conditions and predispose the host to inflammation [42,43].

### 2.2. Probiotics, Prebiotics, and Synbiotics in Microbiota Modulation

While diet exerts a broad ecological impact on the gut microbiome, probiotics directly modulate the microbial community and host physiology, ultimately influencing microbiota modulation. The mechanisms of action of probiotics can be divided into competitive exclusion and immunomodulation. Competitive exclusion limits the colonization of harmful bacteria by allowing probiotics to establish at epithelial adhesion sites or by depleting nutrients available to pathogens. Certain lactobacilli strains synthesize bacteriocins, hydrogen peroxide, or organic acids that suppress enteric pathogens [44,45]. Another mechanism is immunomodulation, where probiotics bind to pattern recognition receptors such as Toll-like receptors to modulate cytokine production, increase secretory IgA, and promote regulatory T cell responses. They also contribute to barrier integrity by increasing the expression of tight-junction proteins. In addition, many conjugated linolic acid and neurotransmitter precursors influence host energy metabolism and may affect brain function [46,47].

Research results reported that probiotic effects are strain-specific and modulated by individual differences in baseline microbiota. According to Szajewska and Kołodziej [48], *Lacticaseibacillus rhamnosus* GG reduces the incidence and duration of antibiotic-associated diarrhea, particularly in children. However, effect sizes vary according to populations and treatment regimens. Also, *Saccharomyces boulardii* has demonstrated efficacy in decreasing recurrence of *Clostridioides difficile* infection when co-administered with antibiotics [49], and in functional GI disorders, *Bifidobacterium infantis* 35,624 has been shown to reduce abdominal pain and bloating in patients with irritable bowel syndrome. However, overall trial outcomes remain inconsistent, and meta-analyses indicate that strain, dosage, and patient subgroup are critical determinants of efficacy [50]. In metabolic health, supplementation with selected lactobacilli and *Bifidobacterium* strains has produced modest improvements in insulin sensitivity, lipid profiles, and body mass index. Nevertheless, reproducibility remains limited across the population. Collectively, these findings demonstrate that probiotic effects are strain-specific and context-dependent, and that inter-individual variation in baseline microbiota significantly influences colonization and functional outcomes [51,52,53].

Prebiotics complement probiotics by selectively promoting the growth and activity of beneficial intestinal taxa, thereby modulating gut microbiota composition and enhancing the production of health-promoting metabolites such as short-chain fatty acids [22]. Representative examples of prebiotics include inulin, fructooligosaccharides, and galactooligosaccharides. Supplementation with these compounds has been reported to increase populations of lactobacilli and *Bifidobacterium* species, elevate butyrate production, and improve bowel regularity [54]. Research results indicate that prebiotic intake reduces systemic inflammatory markers, improves calcium absorption, and modulates lipid and glucose metabolism [55,56,57]. The efficacy of prebiotics, however, is not consistent across individuals. The baseline microbiome largely determines responses. Inulin supplementation, for instance, induces significant bifidogenic effects in individuals characterized by the presence of particular *Bifidobacterium* strains, whereas such effects are minimal in those lacking these strains [58]. This host–microbe interaction highlights the potential for tailoring prebiotic supplementation according to individual microbiome profiles.

Recent research has highlighted synbiotics, which combine probiotics and prebiotics to produce synergistic effects. In complementary synbiotics, each component functions independently. The probiotics colonize the gut and mediate direct physiological effects, whereas the prebiotics promote the growth of resident beneficial taxa. In synergistic synbiotics, the prebiotic is specifically selected to enhance the survival, colonization, and functional activity of the co-administered probiotic strain. Such targeted pairings can support more durable colonization and sustained metabolic benefits (Figure 1) [59]. For example, co-administration of *Bifidobacterium breve* with galactooligosaccharides enhances the persistence of the strain in the infant gut, thereby supporting immune development and providing protection against pathogens [60]. Also, synbiotic formulations have been reported to reduce hepatic steatosis in non-alcoholic fatty liver disease, improve insulin resistance in type 2 diabetes, and ameliorate inflammation in ulcerative colitis [61]. These findings indicate that precise matching of strains and substrates can achieve greater efficacy than probiotics or prebiotics administered individually.

The modulation of the gut microbiome through diet, probiotics, prebiotics, and synbiotics exerts effects that extend beyond the GI tract. In metabolic disorders, dysbiosis characterized by a reduction in butyrate-producing taxa and an expansion of endotoxin-producing species is associated with insulin resistance and systemic inflammation [62]. Interventions that enrich SCFA-producing microbes have been demonstrated to improve glucose tolerance and lipid metabolism. In immune-mediated diseases, including inflammatory bowel disease, microbiome-targeted approaches may restore barrier integrity and attenuate mucosal inflammation, although outcomes are highly strain- and patient-dependent [63,64]. Research mentions that probiotics can alleviate anxiety and depressive symptoms in subsets of patients, while emerging evidence associates synbiotic administration with improvements in cognitive flexibility and stress resilience [65]. Research has demonstrated the systemic significance of microbiome modulation.

### 2.3. Limitation

Although the modulation of the gut microbiome through diet, probiotics, prebiotics, and synbiotics has seen substantial progress, the effectiveness of current approaches remains constrained by persistent limitations. The most problematic limitation is the significant interindividual variability. The same probiotic strain may effectively colonize one host but fail in another, potentially leading to heterogeneous clinical outcomes [51,52]. Microbial viability is also highly susceptible to loss during industrial processing, storage, and GI transit. Also, temperature fluctuations, oxygen exposure, and gastric acidity have been shown to markedly reduce the number of viable organisms that reach the colon [66]. Dosing regimens are generally standardized and inflexible, limiting their ability to account for differences in baseline microbiome composition, dietary background, or health status. Moreover, designing and manufacturing personalized synbiotics is technically demanding because co-formulation must ensure strain–substrate compatibility and maintain multi-strain stability under oxygen, moisture, and temperature stress. These constraints make large-scale production and long-term stability difficult to achieve [24,67,68,69]. Current delivery formats, including capsules, powders, and functional foods, rarely achieve targeted or time-controlled release, which may be essential to optimized effects within specific gut regions.

However, current delivery systems still face persistent challenges, including the rapid loss of probiotic viability during processing and gastrointestinal transit, the limited ability to co-encapsulate multiple strains with compatible prebiotic substrates, and the difficulty in achieving controlled release profiles that align with individual intestinal environments. These unresolved limitations have hindered the development of truly personalized formulations. Three-dimensional food printing offers a distinctive solution by enabling digital control over formulation architecture, spatial composition, and dosage, thereby bridging the gap between microbiome research and personalized nutrition. These limitations underscore the need for advanced delivery technologies capable of preserving microbial viability, enabling precise co-formulation with selected substrates, and allowing customization of formulation structure, release kinetics, and dosage. Three-dimensional food printing is emerging as a promising platform to address these challenges by enabling the digital design and production of personalized probiotic, prebiotic, and synbiotic formulations.

## 3. Three-Dimensional Food Printing for Personalized Nutrition

### 3.1. General Overview of 3D Food Printing

Unlike existing encapsulation or coating approaches that offer limited structural flexibility, 3D food printing directly addresses the unmet needs in probiotic and prebiotic delivery—enabling controllable micro-environments, spatial separation of sensitive components, and on-demand dosage adjustment. Three-dimensional food printing, the application of additive manufacturing principles to edible substrates, allows for the precise and controlled layer-by-layer fabrication of food constructs guided by computer-aided design (CAD) models. In contrast to conventional batch food processing, which generally yields standardized formulations and portion sizers, 3D food printing permits precise modulation of composition, geometry, and texture, thereby providing a framework for personalized nutrition and the development of functional food systems. In this approach, raw materials are reformulated into printable or deposited under digital control to produce a complex structure with high spatial fidelity [70]. Current 3D food printing technologies are commonly categorized into four: extrusion-based printing, inkjet printing, binder jetting, and selective laser sintering. Inkjet printing enables deposition of picolitre-scale droplets and is particularly suited for low-viscosity formulation, including nutrient solutions and flavoring agents, although its applicability is constrained by rheological parameters [71]. Binder jetting uses a liquid binder to solidify a powder substrate, creating a porous structure with relatively flexible properties [72]. Selective laser sintering applies localized laser irradiation to fuse sugar or milk powder particles into intricate three-dimensional constructs, achieving high architectural precision but potentially compromising thermolabile compounds due to localized thermal stress [73]. Finally, extrusion-based printing has been most widely utilized in food applications due to its compatibility with a variety of hydrocolloids, proteins, and complex matrices. In extrusion systems, semi-solid inks are continuously deposited through nozzles to assemble three-dimensional objects in a layer-by-layer manner. Successful fabrication is critically dependent on the rheological properties of the ink [74]. The most crucial aspect of 3D food printing is rheological properties, which ensure both fluidity during printing and stability after printing. In other words, the nozzle must ensure that food ink flows smoothly without clogging, while the structure maintains its defined shape without collapsing. This characteristic is known as shear thinning viscosity, defined as the property where viscosity decreases with increasing shear rate. The following crucial rheological characteristic is yield stress, which represents the minimum stress required for a material to begin deforming. In 3D food printing, a certain level of yield stress is required to prevent the laminated structure from collapsing. However, if the yield stress is too high, the layers will spread, and if it is too low, the food ink will not be output from the nozzle. Therefore, yield stress determines the balance between printability and shape precision. Therefore, active research is underway in 3D food printing to optimize the rheological properties of food inks for different materials [75].

A variety of food-grade biopolymers, including alginate, gelatin, agar, carrageenan, starch derivatives, and proteins, have been extensively employed as matrix materials in printable formulations. These polymers provide tunable viscoelastic properties, undergo gelation through ionic, thermal, or enzymatic crosslinking, and enable encapsulation and stabilization of labile bioactive compounds [76]. Advances in polymer science and process engineering have consequently transformed 3D food printing from a conceptual demonstration into a versatile manufacturing platform with considerable potential for next-generation food design and personalized nutrition.

### 3.2. Three-Dimensional Food Printing for Personalization

Personalization in nutrition requires adapting food composition and functional properties to the specific needs of individuals, reflecting variations in age, physiology, health status, and dietary restrictions. Conventional food processing depends on standardized formulations and large-scale production, which limits the ability to deliver nutrients or functional compounds in a tailored manner. Fortified foods and dietary supplements partially address these gaps, but their formulations remain fixed, with limited flexibility for dosage control, release kinetics, or the incorporation of multiple sensitive bioactives [29].

3D food printing offers a versatile solution to these limitations by combining digital design with precise material deposition. This technology enables the spatial arrangement of nutrients and bioactives into customized structures, allowing not only flexible formulation but also protection and targeted release of functional substances. For example, Oliveira et al. [77] demonstrated that extrusion-printed cookies enriched with encapsulated grape-skin polyphenols exhibited up to 115% higher antioxidant activity and 173% greater phenolic retention than non-printed controls, highlighting the ability of 3D-printed structures to preserve heat- and oxygen-sensitive bioactives during baking. Recent advances highlight the suitability of 3D printing for the incorporation of highly labile bioactives. Yu et al. [78] embedded β-carotene into customized oral films through a digitally controlled printing process, achieving substantial improvements in stability against oxidative and thermal degradation while allowing precise adjustment of dosage. The study further optimized different delivery systems for β-carotene, confirming that 3D printing is an effective platform for extending shelf life and tailoring nutrient delivery. In another study, Park et al. [79] employed coaxial 3D printing to fabricate orodispersible films containing two distinct functional ingredients, curcumin and phycocyanin, within separate layers. Coaxial nozzle 3D printing process enabled simultaneous yet independent control of each bioactive, resulting in enhanced encapsulation efficiency, improved antioxidant stability, and prolonged storage performance. Such approaches exemplify how 3D printing can be leveraged to design multifunctional personalized systems for delivering diverse bioactives.

Current evidence demonstrates that 3D food printing constitutes a transformative strategy for personalized nutrition, particularly in the delivery of functional and bioactive compounds. By integrating formulation flexibility, structural precision, and digital control, this technology enables the manufacturing of foods that are scientifically optimized and individually customized. It thereby addresses the limitations of conventional food processing and establishes new opportunities for implementing personalized diets that combine functional efficacy with consumer acceptance. When integrated with probiotic and prebiotic components, 3D food printing extends beyond conventional personalization by enabling the spatial arrangement and protection of living microorganisms alongside their selective substrates. This functional convergence allows the creation of synbiotic architectures that couple biological efficacy with structural precision, providing a tangible framework for personalized microbiome modulation.

### 3.3. Three-Dimensional Food Printing for Probiotics

The personalized application of probiotics requires precise alignment between strain-specific functionality and the host microbiome context. The efficacy of probiotics depends on strain identity, administered dose, and the physiological conditions under which the cells exert their functions. Conventional delivery formats, such as capsules, powders, and fermented foods, offer limited control over dosage accuracy, release kinetics, and strain viability after manufacture, which restricts the capacity to tailor probiotic functionality to individual microbiome profiles [80,81,82]. In contrast, 3D food printing provides a digital-based approach to customize probiotic products in terms of dosage, structural configuration, and targeted GI delivery. This technology indicates its potential to serve as a digital-based platform for personalized nutrition strategies.

A significant advantage of 3D food printing is the parametric control of structure and dosage. Since edible materials are deposited according to CAD, an identical formulation can be fabricated into distinct geometries and volumes without altering the base matrix. This capacity enables (i) precise titration of viable cell loads across age groups or application contexts, (ii) modulation of surface-to-volume ratios that govern hydration and disintegration dynamics within the GI tract, and (iii) spatial compartmentalization that separates protective layers from cell-containing domains [83]. Such digitally defined constructs facilitate the tailoring of probiotic delivery to host-specific requirements, while it is maintained through deterministic print files. A second advantage is conferred by the capacity to regulate the microenvironment surrounding probiotic cells. Printed matrices can be engineered to modulate physicochemical parameters that are critical for viability and functionality, including transient buffering capacity, water activity, and oxygen exposure, through the spatial distribution of excipients within defined domains of the construct. This microenvironment modulation, digitally specified rather than fixed by bulk processing, enables personalization by embedding the same strain within distinct protective contexts that are tailored to anticipated gastric acidity, bile exposure, or co-ingested dietary components [84,85]. Third, 3D food printing possesses the capacity to enable GI site-specific action in a manner compatible with routine dietary practices. Parameters such as geometry, infill, and layer sequencing can be engineered to regulate disintegration kinetics and intestinal residence time, thereby aligning release profiles with targeted regions of the upper or lower intestine. Unlike conventional pharmaceutical dosage forms, printed foods can incorporate such control into familiar formats, texture, and portion size, an attribute regarded as essential for sustaining long-term efficacy [86,87]. An additional advantage of the technology is its capacity to support data-driven personalization protocols. Printing parameters, ingredient ratio, and target dosages are digitally specified, allowing direct integration with individual-level inputs such as microbiome profiles, dietary patterns, and temporal intake requirements. Within this digital frame formulations can be systematically adjusted for strain composition, dosage, and nutrition balance without the need for reformulation, thereby providing greater adaptability than conventional manufacturing approaches. Although comprehensive integration with dynamic health data remains under active investigation, 3D food printing is increasingly recognized as a promising platform for flexible and personalized probiotic delivery [88,89].

Overall, the integration of digitally defined dosage and geometry, engineered microenvironments, GI site-specific targeting within food matrices, and software-based linkage to individual data establishes 3D food printing as a promising platform for personalized probiotic delivery.

## 4. Advances in Personalized Delivery of Probiotics and Prebiotics via 3D Food Printing

### 4.1. Encapsulation and Targeted Release

**Figure 2:**
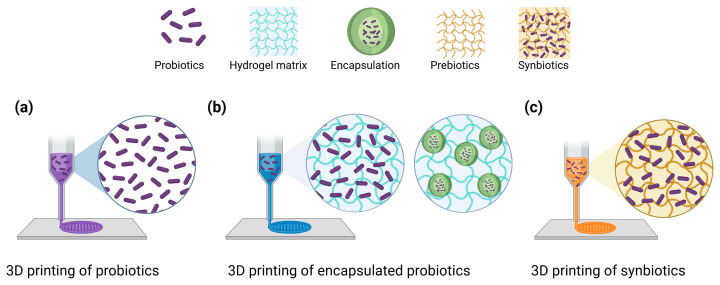
Three-dimensional food printing strategies for probiotics and synbiotics; (**a**) 3D printing of probiotics, (**b**) 3D printing of encapsulated probiotics, and (**c**) 3D printing of synbiotics.

Encapsulation is crucial for the personalized delivery of probiotics in 3D-printed foods, as it preserves cell viability against gastrointestinal stresses. Without protective matrices, most probiotic cells are inactivated by gastric acidity, bile salts, and digestive enzymes before reaching their intended target site (Figure 2a). In contrast, printable structures, especially core–shell and multi-compartment designs, can buffer local pH, reduce proton and bile diffusion, modulate water ingress and disintegration kinetics, and introduce surface functions functionalities such as muco-adhesion to extend intestinal residence, while preserving print fidelity and dosage precision (Figure 2b). Recent studies have demonstrated these principles using food-grade materials and printing modalities that are directly compatible with personalized nutrition (Table 1).

Truong-Le et al. [90] applied coaxial 3D food printing to encapsulate *Bifidobacterium bifidum*. The cells were loaded into a starch-based core and covered with an alginate/pectin shell, forming a pH-sensitive hydrogel. This structure maintained a survival rate of over 96% after printing. In simulated gastric fluid at pH 1.2, all unencapsulated (control) cells were inactivated within two hours, whereas 83.1% of encapsulated cells remained viable, demonstrating strong acid protection. In simulated intestinal fluid at pH 6.8, both encapsulated and free cells showed high survival, each maintaining over 90%. These findings indicate that the 3D-printed core–shell hydrogel can effectively protect probiotics in harsh gastric conditions and shows potential for targeted delivery to the colon. The gel-in-gel system, prepared without coaxial nozzles, also enabled effective delivery of probiotics under varying GI conditions. *Lacticaseibacillus rhamnosus* GG was first encapsulated in alginate microgels and subsequently embedded within a larger gelatin–alginate hydrogel matrix. The microgels contained calcium carbonate, which acted as an acid-neutralizing agent, thereby alleviating acid stress in gastric fluid. As a result, the encapsulated probiotics achieved a survival rate of about 86% after two hours in simulated gastric fluid, compared with near-total loss in the free-cell control that contained no protective matrix. This demonstrates the protective effect of the gel-in-gel structure against acidic environments and supports its potential for targeted probiotics delivery [91].

Recent studies have demonstrated that probiotics can be incorporated into 3D food printing through various encapsulation techniques. This approach protects probiotics from GI stress, thereby enhancing their survival and contributing to improved stability. These findings suggest that the combination of 3D food printing and encapsulation technologies represents a promising platform for personalized probiotic delivery capable of adapting to complex GI conditions.

### 4.2. Printing Conditions and Process Parameters

In extrusion-based 3D food printing, process conditions are not just engineering settings but key factors that determine the thermal and shear stresses experienced by probiotics from the bio inks to the printed structure. Most lactic acid bacteria and bifidobacteria are mesophilic, with fragile membranes and stress-sensitive proteins, so even small increases in temperature or shear can significantly reduce viability during deposition. The geometry of the printed construct, including infill density, filament spacing, and layer arrangement, also shapes internal porosity and surface-to-volume ratio. These structural features influence heat and moisture transfer during drying, storage, and thermal treatment, which in turn define the length and intensity of stress after printing. For example, infill patterns enable faster moisture removal and shorter heat exposure, resulting in higher viable counts than dense patterns baked to the same endpoint. Thus, both nozzle conditions and construct geometry work together to determine probiotic survival. Therefore, the conditions and process parameters of 3D food printing directly influence the final survival of probiotics, and recent studies have experimentally demonstrated these relationships.

#### 4.2.1. Temperature

Liu et al. [92] investigated the influence of printing temperature on the survival of *Bifidobacterium animalis subsp. lactis* BB-12 incorporated into a mashed potato matrix. Their results showed that temperatures of 24–45 °C caused no significant losses, whereas holding the bio-ink at 55 °C for 45 min reduced viable counts from 10.07 to 7.99 log CFU/g, indicating that prolonged residence at elevated temperatures can be particularly detrimental. Consistent trends were observed by Xu et al. [93], who employed a tea-protein-xanthan gum Pickering emulsion as a protective printing ink. In their study, survival was not significantly affected at 45 °C and 55 °C, but exposure at 65 °C for 10 min decreased viability from 8.07 to 6.59 log CFU/g. Together, these findings demonstrate that although encapsulation can protect in simulated GI environments, thermal stress during the printing process remains a critical limiting factor. Accordingly, careful control of temperature parameters, tailored to the tolerance of specific probiotic strains, is essential to maximize cell survival during extrusion-based 3D food printing.

#### 4.2.2. Nozzle Sizes

In addition to temperature, both studies also examined the role of nozzle geometry. Liu et al. [92] reported that extrusion through 1.0 mm and 1.4 mm nozzles caused no significant viability loss. In contrast, use of a 0.6 mm nozzle led to a small but significant reduction (−0.19 log), attributed to higher shear stress and oxygen exposure in narrower tips. By contrast, Xu et al. [93] found no significant differences across the nozzle sizes tested when using the Pickering emulsion gel, suggesting that shear forces did not exceed a damaging threshold under their conditions. These findings indicate that nozzle diameter can influence probiotic survival, but the extent of this effect depends on both the printing matrix and the probiotic strain. Therefore, nozzle size should be carefully optimized when designing probiotic-loaded food inks.

#### 4.2.3. Infill Patterns

In addition, probiotic survival has also been shown to depend on the infill pattern of the printed construct [94]. In this study, cereal-based dough *Lactiplantibacillus plantarum* NCIM 2083 was printed with concentric and honeycomb patterns and then baked. The honeycomb design resulted in approximately 2 log higher survival compared to the concentric pattern. This outcome was attributed to differences in internal structure that influence heat and moisture transfer, with the honeycomb structure moderating local heat penetration and moisture loss.

#### 4.2.4. Post-Processing

Follow-up experiments further demonstrated the impact of post-processing: baking at 145 °C for 8 min reduced viable counts from about 10^9^ to 10^5^ CFU/g, and at 175 °C and 205 °C, even 4 min of baking produced a similar reduction to about 10^5^ CFU/g. These results indicate that shorter baking times at higher temperatures do not improve survival, underscoring that post-processing conditions play a decisive role in determining the fate of probiotics after printing [94]. Probiotic survival is influenced not only by the proven parameters but also by the printing strategy itself. For example, the use of coaxial extrusion can markedly enhance survival. As demonstrated above in the study by Truong-Le et al. [90], *Bifidobacterium bifidum* was encapsulated within a starch-based core surrounded by an alginate–pectin hydrogel shell. Coaxial core–shell printing of Bifidobacterium bifidum maintained more than 96% viability immediately after printing and 83% after 2 h in simulated gastric fluid (pH 1.2), while over 90% of cells remained viable in simulated intestinal fluid (pH 6.8). This high stability demonstrates that dual-layer core–shell architectures effectively buffer acid exposure and support targeted intestinal delivery. Therefore, core–shell configuration provided adequate protection under acidic conditions, resulting in significantly higher survival compared with unencapsulated controls.

According to these studies, probiotic survival in 3D food printing is determined by the interaction between process parameters and printing strategies. Temperature, shear stress, and internal structure each act as factors that can reduce viability, while advanced approaches such as a coaxial nozzle provide effective protection. These findings show that optimizing temperature, nozzle diameter, infill design, and deposition strategy is essential to maintain probiotic functionality. Ultimately, printing conditions and process parameters are not just technical settings but biological determinants, highlighting their critical role in enabling effective and reliable personalized delivery of probiotics.

Most studies to date have examined individual factors—such as temperature, nozzle diameter, internal structure, and post-processing conditions—that influence probiotic viability and printing fidelity. However, in practice these parameters do not operate independently; they interact through complex physicochemical variables such as viscoelasticity, moisture content, heat transfer, and shear stress. For instance, printing temperature and solid concentration simultaneously affect not only viscosity but also the local shear rate within the nozzle, producing combined effects on cell survival and structural stability. Therefore, interpreting the influence of a single variable in isolation provides only a partial understanding of the overall system behavior. Current studies have predominantly focused on one-factor analyses, leaving limited quantitative evidence for interaction effects and insufficient consistency for cross-study comparison. These limitations may explain the variability and occasional contradictions among reported findings, underscoring the need for careful, critical evaluation when interpreting existing data.

## 5. Synbiotic Applications in 3D Printing

Probiotic viability is determined by multiple factors including encapsulation strategies, printing environments and conditions, post-processing treatments, and the physicochemical properties of bio-ink materials. Bioinks must have physical protective properties that help preserve the functionality of loaded functional materials from external stresses and ensure that printed structures remain intact. Additionally, depending on their composition and formulation, bioinks can act as a physical barrier to protect probiotic cells, as well as a nutritional environment, such as prebiotics, that supports metabolic activity during gastrointestinal transit and colonization (Figure 2c) [17,91].

Synbiotics are combinations of probiotics and prebiotics. Probiotics are defined as live microorganisms that confer health benefits on the host, whereas prebiotics are substrates that are selectively utilized by host microorganisms to promote health [24]. Synbiotics are generally classified into two main design strategies, complementary synbiotics and synergistic synbiotics. In complementary synbiotics, probiotics and prebiotics work independently. Probiotics exert strain-specific effects, while prebiotics are intentionally tailored to the metabolic repertoire of the co-administered probiotic strain, enhancing survival, colonization, and functional performance, resulting in an effect greater than the sum of their individual contributions [97,98]. In 3D food printing, selecting the right bioink base is a crucial factor in integrating synbiotics. Prebiotics such as cellulose derivatives, fructooligosaccharides, and maltodextrins can be incorporated into printable matrices to provide appropriate rheological properties and structural stability, while also serving as an organic energy source for the embedded probiotic strains [99]. In other words, bioinks exhibit rheological properties that ensure printability while serving as a protective and nutritive matrix for probiotic cells. Such matrices can mitigate membrane damage during printing, support post-stress recovery, and, when formulated with prebiotic substrates, enable selective fermentation pathways in the colon. Therefore, synbiotics can be effectively implemented through 3D printing [91,100,101].

The digital and programmable nature of 3D food printing allows precise control over parameters such as substrate type and concentration, spatial organization within the construct, and release kinetics. Through this integration, synbiotic formulations manufactured by 3D food printing can be customized for efficient and effective personalized delivery of probiotics.

According to Yoha et al. [95] *Lactiplantibacillus plantarum* NCIM 2083 encapsulated with fructooligosaccharides, whey protein, and maltodextrin retained approximately 98–99% viability during extrusion, confirming that the printing process caused minimal losses. Distinct differences in probiotic survival were observed depending on the post-processing conditions. Constructs produced by spray-freeze drying followed by freeze drying maintained 79% survival and 6.43 ± 0.17 log CFU/mL after 4 h of simulated GI digestion and preserved 96–98% survival with 7.98 ± 0.48 log CFU/mL during 35 days of storage under both refrigerated and ambient conditions. In contrast, free cell controls exhibited 3–4 log reductions during digestion and nearly 2 log reductions during storage. Zhang et al. [96] reported a scaffold-based approach using cellulose hydrogel spiral tubes loaded with *Roseburia intestinalis*, a butyrate-producing commensal species with immunomodulatory potential. The cellulose scaffold functioned both as a physical barrier against gastric acid and bile salts and as a fermentable substrate, extending intestinal retention to approximately 72 h compared with about 12 h for free cells and significantly enhancing the production of butyrate and other short-chain fatty acids. In murine models of high-fat diet induced obesity and inflammatory bowel disease, these synbiotic constructs reduced weight gain, suppressed adipose accumulation, improved lipid metabolism, and attenuated both local and systemic inflammation.

The results indicate that synbiotic 3D food printing can be realized through the material composition of the bio-ink, which integrates both physical protection and metabolic support to enhance probiotic viability and functional activity. Moreover, these approaches highlight the potential of 3D printing to enable precise personalization of functional food, in which synbiotic designs improve probiotic survival, colonization, and metabolic outputs within the GI tract. Importantly, the integration of advanced printing processes such as coaxial or multi-nozzle systems is expected to further expand the design space, allowing more diverse synbiotic formulations to be manufactured with higher precision and efficiency. Ongoing studies are actively advancing these concepts, and this continuous progress supports the expectation the 3D food printing will emerge as a next-generation platform for personalized synbiotic health applications.

## 6. Challenges and Future Perspectives

Although synbiotic 3D food printing presents significant potential, several technical and biological challenges remain to be addressed. The strain-specific and host-dependent variability in probiotic efficacy makes it difficult to develop universally applicable bio-inks and to achieve consistent quality. Outcomes vary markedly among strains and are further influenced by individual differences in gut microbiota composition [85]. Process optimization represents another critical challenge, as the conditions that favor printability often conflict with those required to preserve probiotic viability. While structural fidelity and extrusion efficiency can be improved under elevated temperature or shear stress, such conditions may simultaneously compromise cell survival. Accordingly, ongoing research focuses on developing strategies that balance mechanical stability with biological integrity [93]. The diversity of available bioink materials also remains limited. Most current formulations rely on alginate, gelatin, and a few selected biopolymers as their base components. Therefore, expanding the range of prebiotic substrates and bioactive compounds that can be incorporated into printable matrices, as well as predicting their physicochemical interactions and biological outcomes, remains an important research direction [102]. Beyond these material and process-related constraints, production scalability and cost-effectiveness continue to pose significant barriers to commercialization. Although 3D food printing is well-suited for personalized manufacturing, its relatively slow processing speed and high production cost compared with conventional mass production limit its large-scale industrial application [103]. In addition, from a translational perspective, future research should also clarify how synbiotic 3D food printing can contribute to measurable health outcomes. While most existing studies focus on process optimization and material development, few have evaluated whether printed formulations lead to meaningful modulation of gut microbiota composition or improvement in host metabolic and immune parameters. Establishing such links through controlled human studies will be essential to validate the physiological relevance of printed functional foods. Demonstrating tangible health benefits will not only strengthen scientific credibility but also accelerate regulatory approval and consumer acceptance.

Future advancements depend on the integration of biological fields with cutting-edge technologies. For example, the introduction of coaxial and multi-nozzle deposition systems is expected to enable the simultaneous mixing of multiple bioinks to co-encapsulate probiotics with designed prebiotics or complementary bioactive compounds. Furthermore, the interdisciplinary integration of 3D food printing, microbiome sequencing, and artificial intelligence will further enable real-time personalization [104]. In this approach, individual microbiome profiles can be translated into optimized strain and substrate combinations, and the resulting formulations can be reproduced as digitally defined printable recipes. Furthermore, emerging concepts such as 4D and 5D printing may advance synbiotic constructs that dynamically modify their physicochemical properties, including texture, color, and nutritional value, in response to fermentation or other time-dependent transformations, thereby enhancing functional efficacy [105].

In addition to these technical and biological limitations, several regulatory, ethical, and economic challenges must also be resolved to translate synbiotic 3D food printing into practical applications. Regulatory frameworks for personalized and printed foods remain nascent and fragmented across regions, with limited guidance on the classification, microbial safety assessment, and labeling of 3D-printed functional products. In particular, probiotic-containing matrices require strain-level validation and dose-specific stability testing under simulated gastrointestinal conditions before clinical endorsement, yet current approval pathways were not designed for digitally fabricated or on-demand food products. Ethical considerations also arise from the use of individual microbiome sequencing data to design personalized formulations. Issues of data privacy, informed consent, and equitable access to algorithm-driven dietary recommendations remain under debate. Economically, the high cost of bioink ingredients and limited throughput of extrusion-based systems constrain scalability. Cost analyses indicate that material utilization efficiency, multi-nozzle automation, and recyclable biopolymer systems will be critical to achieving cost parity with conventional nutraceutical manufacturing. Therefore, establishing harmonized regulatory standards, ethical data governance, and sustainable production frameworks will be essential for the responsible commercialization of synbiotic 3D-printed foods.

In summary, the future of synbiotic 3D food printing will rely not only on improving biological protection and probiotic viability but also on achieving technological sophistication, personalization, and scalable productivity. Equally important will be the establishment of coherent regulatory frameworks, ethical data governance, and economically sustainable production systems that ensure consumer safety, transparency, and accessibility. Only through such multidisciplinary integration can synbiotic 3D-printed foods progress from laboratory feasibility to real-world adoption in personalized nutrition. Moreover, the long-term success of synbiotic 3D-printed foods will ultimately depend on consumer acceptance and behavioral adaptation. As personalized food technologies move from laboratory research to market implementation, understanding public perception, sensory expectations, and trust toward digitally fabricated foods will be critical. Addressing these social and behavioral dimensions through transparent communication, education, and evidence-based validation will help bridge the gap between technological innovation and its real-world adoption in personalized nutrition.

## 7. Conclusions

Three-dimensional food printing represents a transformative platform for the delivery of probiotics, prebiotics, and synbiotics. This technology is being cited as a way to overcome the limitations of existing methods in the field of personalized medicine by implementing a digital structure system that integrates structural stability, microbial stability, and release control functions. Furthermore, the use of 3D printing in the microbiome is considered a promising tool for efficient and precise nutrition, as it improves strain-to-strain variability, gastrointestinal stress tolerance, and long-term storage stability. This is a convergent field that connects microbiome science, food engineering, and personalized nutrition, and recent studies are proposing an integrated approach that combines 3D food printing and microbial technologies to enhance microbial viability. Encapsulation strategies, material properties, printing parameters, and synbiotic combinations are being actively researched to find optimal conditions for each characteristic. Research is currently underway to integrate 3D food printing with advanced printing processes such as microbiome profiling, AI-based formulation, and coaxial and multi-nozzle deposition. These developments are expected to positively impact the field of personalized microbiomes by supporting the development of highly personalized synbiotic foods that not only maintain viability during processing and storage, but also actively modulate microbial ecology and host physiology. Ultimately, the convergence of digital manufacturing and microbiome-based nutrition is expected to fundamentally reshape the paradigm of functional food development and drive the next generation of personalized dietary strategies.

## Figures and Tables

**Table 1:** Personalized probiotics and synbiotics via 3D food printing.

Type	Strain	Design	Matrix/Scaffold	Key Process Parameters	Outcomes-GI	Outcomes–Storage/In Vivo	Note	Ref
Probiotics	*B. bifidum*	Coaxial core–shell	· Core: starch · Shell: alginate + pectin (pH-responsive)	· Post-printing survival > 96%	· SGF pH 1.2 (2 h) : 83.1% · SIF pH 6.8: >90%	-	Strong gastric acid protection Potential for targeted intestinal delivery	[90]
	*L. rhamnosus* GG	Gel-in -Gel	· Alginate microgels (with CaCO_3_) in gelatin–alginate matrix	· Print ~30 °C · 4–5 layers	· SCF 2 h: ~86% (encapsulated)	-	CaCO_3_ buffering and microenvironmental protection	[91]
	*B. animalis subsp. lactis* BB-12	Extrusion	· Mashed potato ink	· 24–45 °C: ns · 55 °C (45min) · Nozzle: 0.6/1.0.1.4 mm	-	· 55 °C (45 min) : 10.07→7.99 log CFU/g · 0.6 mm: −0.19 log	Prolonged high temperature and narrow nozzle (shear/oxygen) are detrimental	[92]
	*B. animalis subsp. Lactis*	Extrusion (pickering)	· Tea protein + Xanthan gum	· 45–55 °C: ns · 65 °C (10 min)	-	· 65 °C : 8.07→6.59 log CFU/g · Nozzle effect: ns	Thermal-stress threshold identified	[93]
	*L. plantarum* NCIM 2083	Extrusion Infill + baking	· Cereal (wheat) dough	· Infill : Honeycomb vs. concentric · Baking 145–205 °C	-	· Honeycomb: 10^7^ · Concentric: 10^5^ · Baking: ~10^9^→10^5^ CFU/g	Internal geometry and baking conditions govern survival	[94]
Synbiotics	*L. plantarum* NCIM 2083	Encapsulation + extrusion	· Fructo-oligosaccharides, whey protein, maltodextrin	· Spray freeze + freeze dry	· 4 h GI : 79%, : 6.43 ± 0.17 log CFU/mL	· 35 d (4 °C & ambient) : 96–98% : 7.98 ± 0.48 log CFU/mL · Free: GI −3~−4 log · Storage: ~−2 log	Prebiotics serve both protective and nutritive roles	[95]
	*Roseburia intestinalis*	Cellulose spital tube	· Cellulose hydrogel (barrier + fermentable substrate)	· Oral delivery	-	· Intestinal retention ~72 h vs. ~ 12h (free) : butyrate/SCFAs ↑ · In HFD/IBD mice : Weight/adiposity ↓ : lipids ↑ : inflammation ↓	Scaffold provides simultaneous physical protection and metabolic support	[96]

## Data Availability

No new data were created or analyzed in this study. Data sharing is not applicable to this article.

## Abbreviations

The following abbreviations are used in this manuscript:

3D	Three dimensional
SCFA	Short-chain fatty acid
CAD	Computer-aided design
CFU	Colony Forming Unit
GI	Gastrointestinal
SGF	Simulated gastric fluid
SIF	Simulated intestinal fluid
AI	Artificial intelligence
